# The ethical dilemma of ventilator sharing during the COVID-19 pandemic

**DOI:** 10.7189/jogh.10.020392

**Published:** 2020-12

**Authors:** Harshil Bhatt, Sandeep Singh

**Affiliations:** 1 Goshen Hospital, Goshen, Indiana, USA; 2 Indiana University School of Medicine, South Bend, Indiana, USA

Coronavirus disease 2019 (COVID-19) pandemic has led to a significant shortage of medical supplies, including Personal Protective Equipment (PPE), ventilators, and extracorporeal membrane oxygenation machines (ECMO). The demand and supply ratio of these equipment has gone out of control in many hardest-hit COVID-19 parts of the world. From a health care point of view, the chaos created by this pandemic has compelled health care professionals to take unprecedented and difficult steps to allow the proper allocation of these resources to save lives of those affected by the virus and to also protect the vulnerable population from contracting the virus. The decision to allocate ventilators to critically ill patients, in particular, has been the toughest decision for health care professionals to make due to its ethical complexity. Similarly, the proposal for using a single ventilator on more than one patient has been met with significant ethical challenges.

The idea of sharing ventilators on patients is born out of the agony of health care professionals to avoid denying care to their patients and saving their lives. We, as health care professionals, are bound to the “Hippocratic Oath”, the very founding pillar of our medical education and health care system. “*Primum non nocere*” (“First, do no harm”), is the ethical principle of modern medicine. However, uncertainty takes birth when deciding to share the same medical equipment for more than one patient.

So, why is it so ethically arduous to allocate one ventilator to more than one patient? The concept of ventilator sharing was initially described by Paladino et al. in 2007, where they used a shared ventilation system to successfully ventilate four adult-human-sized sheep for twelve hours [1]. A laboratory experiment was conducted by Branson et al. in 2011 to ventilate four artificial lungs through a single ventilator system in which they found that there were vast variations in the amount of tidal volume that was delivered to each subject, and the airway pressure, volume, and flow parameters could not be individualized, leading them to recommend against the use of this method [2]. Due to the diverse respiratory mechanics of each individual patients in acute respiratory failure, it is paramount to individualize ventilatory support. Still, there has been no concrete scientific data available yet that has assessed the outcome of shared ventilation in humans. It is our fundamental duty, as health care providers, to practice medicine based on scientific merits and moral principles. Moreover, health care providers could encounter several conflicts during the process of providing shared ventilation that could question its underlying morality. For instance, selecting two COVID-19 patients who have a similar cardiopulmonary function so that proper ventilator settings can be applied to all, is somewhat tricky. Selected ventilator settings may not be adequate for patients sharing the same ventilator. Also, selecting patients who have a comparable probability of a good outcome or survival from utilizing a ventilator is equally essential, something that cannot be estimated easily in a crisis.

Acquiring informed consent for ventilator sharing could be problematic as well. Patients and families should be made aware that this is not a proven standard-of-care treatment. Moreover, how would one respond to the patients' and their family members' inquiries concerning the other patient or patients receiving support from the same ventilator? Should the sharing of patient information be allowed? This creates another ethical conundrum since releasing protected health information (PHI) could harm patient privacy and violate Health Information Portability and Accountability Act (HIPPA) rules. Besides, it might also be true that all such processes could be deemed as time-consuming and may not be carried out most comprehensively during a time of crisis.

While one is depriving a patient of standard-of-care to treat their respiratory failure, are they also unknowingly being exposed to potential infections that could lead to dire consequences? Also, how can it be known when a clinical condition deteriorates that it was due to being on a shared ventilation system? These are questions that are not easy to answer but need to be strongly considered.

We can see that all the above conflicts pose a serious threat to the ethical structure of providing proper medical treatment. Therefore, the biggest ethical dilemma of ventilator sharing is compromising care to save more lives. From what would be a lifesaver for a single patient could end up being a severe treatment failure, risking the lives of all the involved patients.

**Figure:**
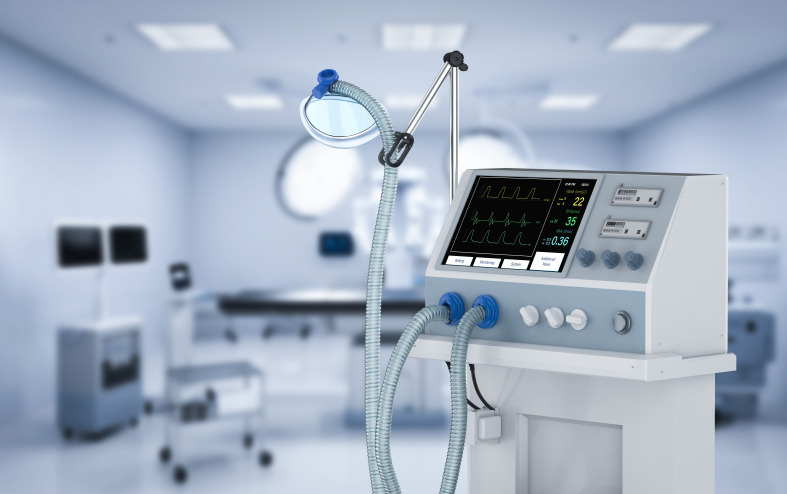
Photo: By Phonlamai for depositphotos (used with permission).

It was not surprising that Society of Critical Care Medicine, American Society of Anesthesiologists, Anesthesia Patient Safety Foundation, American Association of Critical-Care Nurses, and American College of Chest Physicians issued a joint statement in March 2020 to advise clinicians to refrain from using shared ventilation due to medical and ethical implications [3]. Although, as a part of preparing for the surge of COVID-19 patients, many health care centers who have developed their ethical ventilator allocation protocols have also formulated protocols for ventilator sharing [4]. However, without proven medical research to show the survival benefits of utilizing a shared ventilation system and without the availability of better technology that could allow such sharing, it will always be an ethically onerous task for health care providers to implement it. At this point, even doing research involving COVID-19 patients with respiratory failure to explore benefits of ventilator sharing could have ethical problems.

Our cognizance of this problem and the experienced learning from treating the most critical patients during the COVID-19 pandemic could take us a long way in our journey to ethically deliver the best health care in the most challenging times, while faithfully adhering to “*Primum non nocere!*” Till then, the quest to answer the following will go on – what could be more harmful – denying care or compromising care?
